# Using consumer-wearable technology for remote assessment of physiological response to stress in the naturalistic environment

**DOI:** 10.1371/journal.pone.0229942

**Published:** 2020-03-25

**Authors:** Serguei V. S. Pakhomov, Paul D. Thuras, Raymond Finzel, Jerika Eppel, Michael Kotlyar

**Affiliations:** 1 Department of Pharmaceutical Care and Health Systems, College of Pharmacy, University of Minnesota, Twin Cities, Minnesota, United States of America; 2 Department of Psychiatry, University of Minnesota, Twin Cities, Minnesota, United States of America; 3 Department of Experimental and Clinical Pharmacology, College of Pharmacy, University of Minnesota, Twin Cities, Minnesota, United States of America; Institute of Clinical Physiology (IFC), National Research Council of Pisa, ITALY

## Abstract

Psychosocial stress is a major risk factor for morbidity and mortality related to a wide range of health conditions and has a significant negative impact on public health. Quantifying exposure to stress in the naturalistic environment can help to better understand its health effects and identify strategies for timely intervention. The objective of the current project was to develop and test the infrastructure and methods necessary for using wearable technology to quantify individual response to stressful situations and to determine if popular and accessible fitness trackers such as Fitbit® equipped with an optical heart rate (HR) monitor could be used to detect physiological response to psychosocial stress in everyday life. The participants in this study were University of Minnesota students (n = 18) that owned a Fitbit® tracker and had at least one upcoming examination. Continuous HR and activity measurements were obtained during a 7-day observation period containing examinations self-reported by the participants. Participants responded to six ecological momentary assessment surveys per day (~ 2 hour intervals) to indicate occurrence of stressful events. We compared HR during stressful events (e.g., exams) to baseline HR during periods indicated as non-stressful using mixed effects modeling. Our results show that HR was elevated by 8.9 beats per minute during exams and by 3.2 beats per minute during non-exam stressors. These results are consistent with prior laboratory findings and indicate that consumer wearable fitness trackers could serve as a valuable source of information on exposure to psychosocial stressors encountered in the naturalistic environment.

## Introduction

Exposure to frequent, sustained or severe stressors has been associated with the development or progression of disease in multiple therapeutic areas including cardiovascular disease [1], type 2 diabetes [2], obesity [3], sleep disorders [4,5], depression [6], stroke [7] drug addiction (including opioid, tobacco, cannabis and cocaine use) [8–12] and Alzheimer’s disease [13]. The significance of stress has been perhaps best studied with respect to its effects on cardiovascular disease with studies finding increased risk of events associated with exposure to stressors commonly encountered in life such as work stressors, anger episodes and even viewing a stressful sporting event [14–16].

The physiological response to stress is a biological process that is characterized by activation of the sympathetic system as reflected by increases in blood pressure, heart rate, plasma epinephrine concentrations and skin conductance and activation of the hypothalamic-pituitary-adrenal axis as reflected by increases in corticotropin-releasing factor and cortisol concentrations [17–27]. These processes, by which the body responds to stressful events, has been referred to as allostasis–increased allostatic load can lead to pathophysiology which subsequently can result in altered response to and recovery from future stressors [28–31]. Assessing stress response typically occurs in laboratory settings in which participants are exposed to a standardized stressor while physiological parameters are monitored. These laboratory stressors are expensive to administer and burdensome for participants and, therefore, cannot be completed in large numbers of individuals.

Assessing exposure to stressful situations is typically accomplished by either asking individuals about stressful events that have occurred in the past or collecting information about events as they are occurring (or shortly after they have occurred) using Ecological Momentary Assessment (EMA) methods [32–34]. Questionnaires asking about stressful events in the past are subject to recall bias in that individuals may not be able to completely or accurately recall events that have occurred previously. EMA methodologies are effective but somewhat intrusive in that participants are expected to fill out questionnaires multiple times per day in order to accurately capture events in close to real time. EMA studies are therefore usually limited to several weeks as it would be difficult to maintain compliance for prolonged periods of time.

In order to understand how acute stress response measured in the naturalistic environment can translate into chronic disease intervention it is necessary to be able to quantify multiple factors including the magnitude of an individual’s response to stress, the pattern of recovery, and the frequency of stress exposure [29,35]. While these factors may lend themselves to be measured using wearable devices, past stress history also needs to be considered along with these measurements as prior history of stress exposure may either attenuate or exaggerate the observed acute stress responses [28,29]. Currently available methods to assess both when an individual is exposed to a stressor (i.e., stress exposure) and the magnitude of the physiological response to that stressor (i.e., stress response) have significant limitations as described above.

Accessible consumer wearable sensor technology already accepted by a wide range of individuals provides an ideal platform for scalable approaches to measuring stress exposure in large populations. However, this technology is fairly new and requires extensive investigation to determine its usefulness and reliability for this purpose. There are two particularly challenging aspects to this approach. First, it is necessary to develop informatics tools for obtaining and processing large quantities of time-series data from wearable devices. Second, it is necessary to develop and validate methods for using naturally occurring stressors and artificial but standardized stressors to be used as benchmarks. A number of studies have begun to examine the use of wearable technology for healthcare applications such as the prediction and prevention of falls in the elderly [36], the capture of mental and behavioral events (craving, stress and mood) associated with illicit drug or tobacco product use [37,38], and the identification of activity pattern changes in everyday life [39]. Groups such as the Center of Excellence for Mobile Sensor Data-to-Knowledge initiative [40] have been established to develop appropriate methods for collecting and analyzing data from wearable devices. Studies focusing on college students have used data collected from wearable devices, mobile devices and electronic diary questionnaires to determine overall perceived levels of stress or well-being [41–43]. Previous studies however have not extensively investigated the use of commercially available, relatively low cost, commonly used devices in order to identify stressful periods experienced in the naturalistic environment. Developing methods with commonly used and accepted devices would allow stress exposures and response to be measured remotely and in a large number of individuals enabling the collection of data for stress related research from a much higher number of individuals than could otherwise be obtained.

The objective of the current study was to determine if popular and accessible fitness trackers such as Fitbit® equipped with an optical heart rate monitor could be used to detect physiological response to psychosocial stress in everyday life and if such a study could be conducted without the need for in-person clinic visits. Our hypothesis was that participants’ heart rate would be measurably elevated during self-reported stressful episodes as compared to an individually determined baseline.

## Materials and methods

This minimal risk study was conducted at the University of Minnesota and was approved by the Institutional Review Board.

### Study design

**Fig 1:**
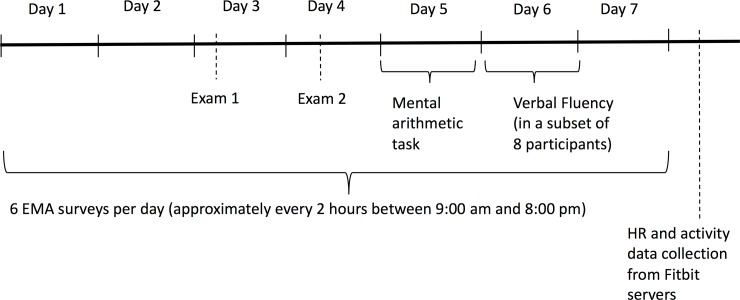
Study outline example for participants that had an exam on days 3 and 4 of the observation period.

In this study, University of Minnesota students who owned a Fitbit® device capable of measuring heart rate and who indicated that they had upcoming examinations were enrolled in a study in which they were asked: 1) for a seven day period to wear their Fitbit® during all waking hours; 2) for a seven day period to complete short surveys six times daily in which they were asked about the occurrence of stressful life events; 3) to once during the seven day period complete a mental arithmetic task over the telephone (while wearing their FitBit®); and 4) for a sub-population of participants to once during the seven day period complete a verbal fluency task over the telephone (while wearing their FitBit®). All interactions with study participants took place online either via web interfaces, an interactive voice-response telephone system, or email. An example of the study timeline for a participant that had an exam on days 3 and 4 is shown in Fig 1.

### Participants

The participants in this study were University of Minnesota students who were at least 18 years old, owned a Fitbit® activity tracker capable of measuring heart rate (i.e., was equipped with a photoplethysmography sensor), and had at least one upcoming examination in a course that they were taking. Potential participants were excluded if they regularly used tobacco products (tobacco is known to increase heart rate), were pregnant or breastfeeding, had an unstable medical condition or were taking medications known to affect heart rate. Participants were also excluded if, at the time of the initial eligibility screening, we were unable to access their Fitbit® data for any technical reason that could not be resolved. All other inclusion / exclusion criterion were based on self-report. Those who completed the entire study were provided with a $50 gift card.

#### Study procedures

**Fig 2:**
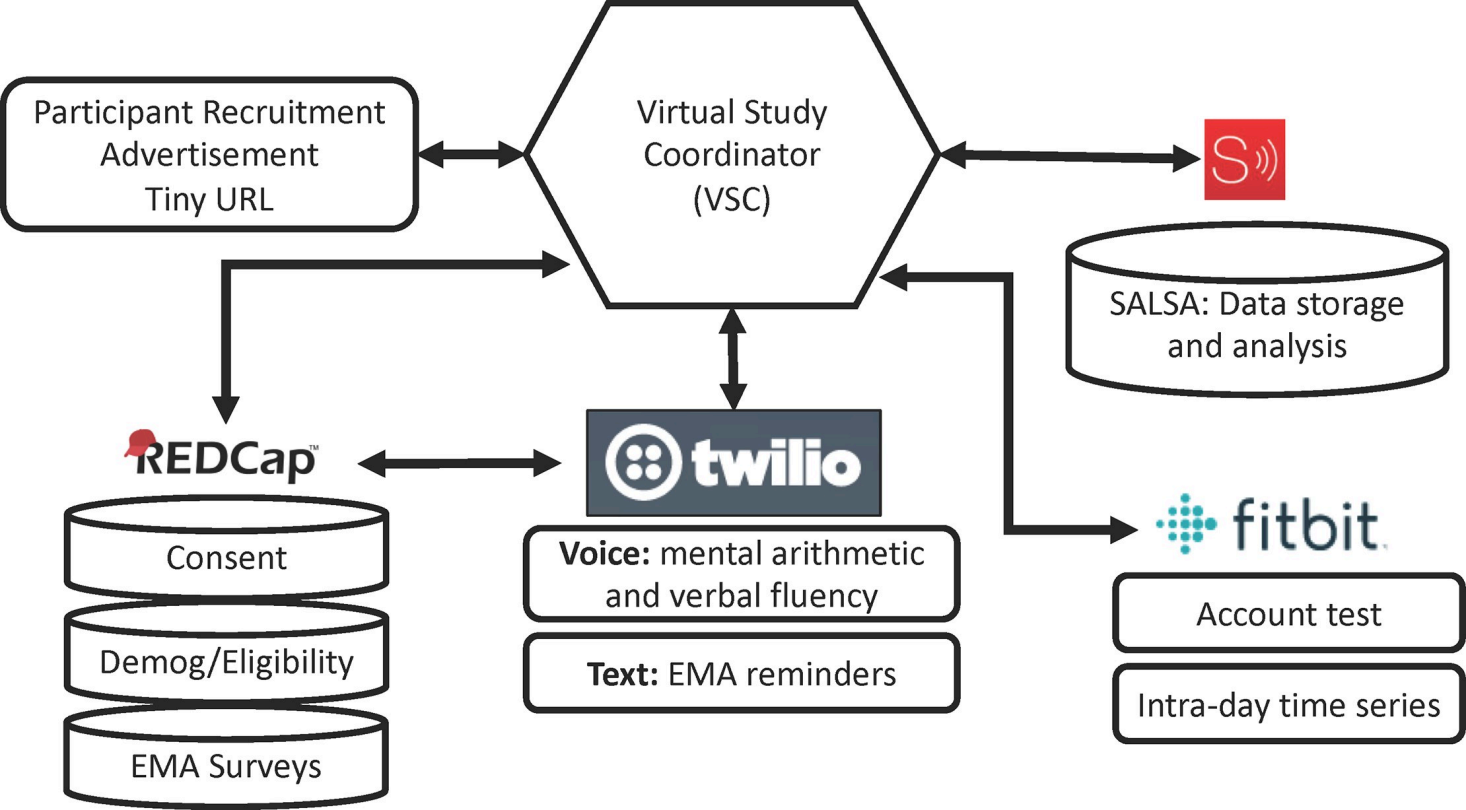
Infrastructure created to obtain and analyze data from wearable devices.

The overall framework of the informatics infrastructure that was created for this pilot study is illustrated in Fig 2. The design of the framework is centered on the concept of a computer application (https://github.com/UMN-RXInformatics/virtual-study-coordinator.git) that acts as a Virtual Study Coordinator (VSC), responsible for orchestrating various study procedures and interactions between systems. Although, as described below, some of the procedures were performed manually in this pilot study, they do lend themselves well to automation in future larger studies. No in person visits were conducted in this study.

Potential participants responded to advertisements posted around the University by following a web link provided in the posting that took them to a consent form. Those who electronically provided informed consent were automatically directed to a screening survey to determine their eligibility for the study. The informed consent and the eligibility survey were implemented using the University of Minnesota Clinical and Translational Science Institute’s REDCap system. After completing the eligibility survey, participants were automatically directed to a web app designed for obtaining Fitbit® data. Compliant with the Fitbit® authorization requirements and procedures, the web app redirected the participants to the Fitbit® login and authorization page on which participants were asked to share with the study their intraday heart rate and activity data collected over the previous 24-hour period. These data were not used in the analysis and were collected as part of screening to ensure that participants’ data were accessible.

Study eligibility and the start date of the 7-day study observation period were determined manually by the study team. The start date was chosen so as to capture as many stressful exams as possible (as indicated by the participants on the screening questionnaire) during the 7-day observation period.

Eligible participants were contacted by email to inform them of their eligibility, to provide them additional study information and to ask them to wear their Fitbit® tracker during all waking hours for all 7-days of the observation period. Participants were asked to charge their Fitbit® devices regularly to ensure their continuous operation. A reminder email was sent on the day immediately prior to the start date of the observation period.

In order to determine the presence of any stressful events that occurred and the time of their occurrence, participants were sent a survey 6 times daily at 132-minute intervals between the hours of 9:00 AM and 8:00 PM each day of the 7-day observation period. Participants had 2 hours to respond to each of these Ecological Momentary Assessment (EMA) surveys. In order to maximize survey response, part of the participants’ compensation was contingent on completing at least 80% of surveys within the 2 hour period.

In order to determine if a standardized stressor delivered remotely could be used effectively to increase heart rate to an extent measurable by a FitBit®, participants approximately half-way through the 7-day period were contacted by email to ask them to make a phone call to an automated telephone system to complete the mental arithmetic task. A subset of 8 of the 18 participants were sent an email on the subsequent day to ask them to make another call to an automated telephone system in order to complete two verbal fluency tasks. In both cases, participants were asked to call the number as soon as possible to complete the tasks.

### Instruments

#### Surveys

The EMA surveys that participants completed 6 times daily during the observation period asked if any stressful events were experienced since the previous EMA. Those who responded in the affirmative were asked how many stressful events were experienced, to indicate the time of the most stressful event, the type of stressor that caused it (i.e., exam in my class, other work / school, interpersonal, financial, health, trauma, other) and how stressful they perceived the event to be on a variable analogue scale anchored by “not stressful at all” and “extremely stressful”. The timing and delivery of EMA surveys was managed by the University of Minnesota Research Electronic Data Capture (REDCap) system enabled with the Twilio text messaging service. REDCap is a secure, web-based application designed to support data capture for research studies [44].

#### Standardized stressor tasks

All participants in this study were asked to complete a modified version of the standardized mental arithmetic task from the Trier Social Stress Test [45]. In this task, participants were asked to continuously subtract the number seven starting from 900 for 2 minutes. This task was implemented on the Twilio telephony platform as a series of scripts written in Twilio Markup Language (TwiML). The participants used their phone’s keypad to enter their responses to the task following automated prompts. If an incorrect answer was entered, the participant was informed that the answer was incorrect and was asked to re-enter the correct response.

A subset of participants were also asked to complete another telephone-based cognitive task (i.e., a verbal fluency test) in which they were asked to first name all words they could think of in one minute that started with the letter “A” (excluding proper names and morphological variants) and then name all animals they could think of also in 1 minute. In prior work, heart rate was found to be negatively correlated with performance on the letter fluency task suggesting that this task is associated with sympathetic arousal [46]. In both tasks, participants were informed that their responses were being recorded. All responses were recorded and stored using the System for Automated Language and Speech Analysis (SALSA: [47,48]).

#### Fitbit® web app

A web-based app was created specifically for this study that was designed to authenticate study participants with Fitbit® and transfer their intraday heart rate and activity (steps and elevation) data for the 7-day study observation period to the SALSA system for storage and analysis. Permission to access intraday time-series data for study participants was obtained from Fitbit® prior to the study. Data were obtained with the highest granularity available via the Fitbit® API (1 second resolution for heart rate data and 1-minute resolution for steps and elevation). Due to how the Fitbit® device estimates and reports heart rate data, setting the 1-second resolution does not guarantee receiving heart rate estimates at this exact resolution. The actual resolution varied from 1 to 15 seconds with the majority of measurements around a 3–5 second interval.

### Data analysis

#### Pre-processing

HR time series were filtered to exclude samples obtained during periods of physical activity (more than 10 steps per minute or 1 or more floors of elevation). The excluded samples were used to calculate HR during increased physical activity (see comparison C1 in the next section). Stressful periods as determined from EMA surveys were defined in terms of 20 minute windows (10 minutes prior to and post the time of the stressor indicated by the participant). Stressful periods during exams reported at baseline were defined as the first 20 minutes of the scheduled exam. The choice of the window sizes is motivated by prior work by Gjoreski et al. (2017) that reported better stressful event prediction accuracy with smaller windows of 10–18 minutes prior and after the reported event. Stressful periods for the remote arithmetic and verbal fluency tasks were defined as the period from when the call was placed to when it was completed. All data samples from EMA periods reported as stress-free and did not have corresponding steps and elevation activity were used to calculate the baseline HR.

#### Within subject comparisons

To compare periods of stress (EMA stress, exam, verbal fluency, and mental arithmetic task) with EMA reported stress-free periods and with periods with increased physical activity (exertion) we defined the following 7 within-subject comparisons (C1-C7) based on the window during which HR measurements were collected:

- C1 –HR measurements collected when physical activity (> 10 steps per minute or > 1 floor of elevation) was present within approximately 2 hour EMA windows reported as stress-free
- C2 –HR measurements collected during 20 min window centered on time of EMA stressor
- C3 –HR measurements collected during approximately 2 hour EMA window reported as containing a stressful event
- C4 –HR measurements collected during 20 min window centered on time of exams reported on EMA
- C5 –HR measurements collected during first 20 min window of exams reported during screening
- C6 –HR measurements collected during Verbal Fluency task
- C7 –HR measurements collected during Mental Arithmetic task

The baseline HR was calculated from frames with no physical activity present (< = 10 steps per minute or < = 1 floor of elevation) collected within approximately 2-hour EMA windows reported as stress-free.

### Statistical analysis

To analyze the within-subject comparisons listed (C1-C7), we employed mixed effects models to compare mean HR estimates during each type of stressful period to periods marked as not stressful. These models clustered HR readings within target periods and within subject. Measurement period (e.g. exam vs. EMA stress free) was a fixed effect and the intercept was modeled as a random effect. The use of mixed effects models ensured that appropriate baseline means were calculated for comparisons in subsets of participants due to within-subject clustering. This multilevel analysis had sufficient statistical power to detect relatively small differences (< 1 heart beat per minute) in heart rate within subject.

To examine the correlation between ratings of stressful events and HR response to stressful events we used the mean HR change for each event and the corresponding rating of that event. We used mixed models to account for the non-independence of ratings of multiple stressful events within individuals.

Comparisons between the mean stressfulness ratings of the upcoming exams during the screening phase of the study to those reported on EMA assessments after individual exams had taken place were tested using the paired Student’s t-test. Significance threshold was fixed at alpha = 0.05. Correlations between stress ratings were tested using Spearman rank correlation.

## Results

### Study sample characteristics

Seventy-six potential participants responded to the study advertisement. Of these, 22 either did not answer the consent form comprehension question or answered it incorrectly and were disqualified. Of the remaining 54 participants, 34 did not meet the eligibility criteria. The remaining 20 participants initially met the eligibility criteria; however, during screening, we were not able to obtain Fitbit® data for technical reasons from 2 of these participants. Thus, the final study sample consisted of 18 participants (mean age 20.06 (SD 2.04) years old; 14 women and 4 men). Of the 18 participants, 1 participant indicated that they had 8 upcoming exams, 4 participants had 5 upcoming exams, 3 participants had 4 upcoming exams, 4 participants had 3 upcoming exams, 5 participants had 2 upcoming exams and 1 participant had 1 upcoming exam. The mean self-reported stressfulness (measured with VAS 0–100) of the upcoming exams that the participants indicated during screening was 63.7 (SD 13.27). The mean self-reported stressfulness of the exams reported post exams on EMA assessments was 72.4 (SD 16.57). The difference between the stress ratings on screening and EMA was not significant (p = 0.097) and individual ratings provided during screening and on EMA were not correlated (rho = 0.25, p = 0.345).

From these 18 participants a total of 837 EMA surveys were requested. Eighty nine (10.6%) of these surveys were not completed. Only one participant completed all surveys. Three participants accounted for half (n = 48) of the 89 incomplete surveys. The number of missed EMAs varied by time of day from 12 for the last EMA of the day to 22 for the first EMA of the day. Of the remaining 747 completed surveys, 73 (9.7%) reported a stressful event had occurred. Exams (27.4%) and work / school stressors (30.1%) were the most commonly reported stressors followed by interpersonal (13.7%), other (13.7%), health (12.3%) and trauma (2.7%).

A subset of 8 of the 18 participants were asked to complete the verbal fluency task in addition to the mental arithmetic task. All 8 completed the task. Of the 18 participants that were asked to complete the mental arithmetic task, 13 (72%) completed the task and 5 did not.

### Heart rate changes

A total of 1,928,738 heart rate (HR) measurements were obtained for all participants. After excluding measurements that coincided with changes in elevation or 10 or more steps per minute, 1,566,238 HR measurements remained. Based on EMA data, 656,700 HR measurements were obtained in stress-free periods and 156,467 measurements within the two-hour window for a stressful event but not in the 20-minute acute stress period, and 21,513 HR measurements within the 20 minute EMA reported stressful event period. For the verbal fluency task, we obtained a total of 275 HR measurements that represented 8 participants. For the mental arithmetic task, we obtained 496 HR measurements that represented 13 participants.

The analysis of HR changes in comparisons C1-C7 from baseline using mixed effects modeling (summarized in Table 1) showed that during exam periods based on participant’s schedules obtained during screening (comparison C5), HR was significantly elevated by 3.90 beats per minute (F(1,668745.7) = 922.3, p<0.001), and during all EMA periods (other than exams) self-reported by participants as stressful (comparison C2), heart rate was significantly elevated by 3.16 beats per minute as compared to stress-free periods (F(1, 673122) = 739.2, p<0.001). When the stressful event was identified as an exam on the EMA survey (comparison C4), HR was elevated by 8.86 beats per minute compared to stress-free periods (F(1,665648) = 2620.5, p<0.001). As expected, during physical activity (comparison C1), the HR was also significantly elevated by 18.63 beats per minute (F(1,240725) = 2158.2, p<0.001)).

**Table 1:** Estimates in heart rate change during various comparisons. The change in HR is relative to the baseline HR as estimated by mixed effects modeling (baseline HR = 76.7, 95% CI = 72.8–80.6) calculated from 2-hour EMA windows reported as stress-free with no physical activity.

Experimental condition	Number of participants	Estimated change from baseline in mean HR beats per minute	95% CI	p
C1. 2 hour EMA windows reported as stress-free (with physical activity)	18	18.63	18.55–18.71	< .001
C2. 20 min window centered on time of EMA stressor (excluding exams)	18	3.16	2.94–3.39	< .001
C3. 2 hour EMA window reported as containing a stressful event	18	.917	.84-.99	< .001
C4. 20 min window centered on time of exams reported on EMA	18	8.86	8.52–9.20	< .001
C5. first 20 min of exams reported during screening	18	3.90	3.65–4.15	< .001
C6. Verbal Fluency task	8	7.09	5.53–8.66	< .001
C7. Mental Arithmetic task	13	-.57	-1.73- .60	.34

**Fig 3:**
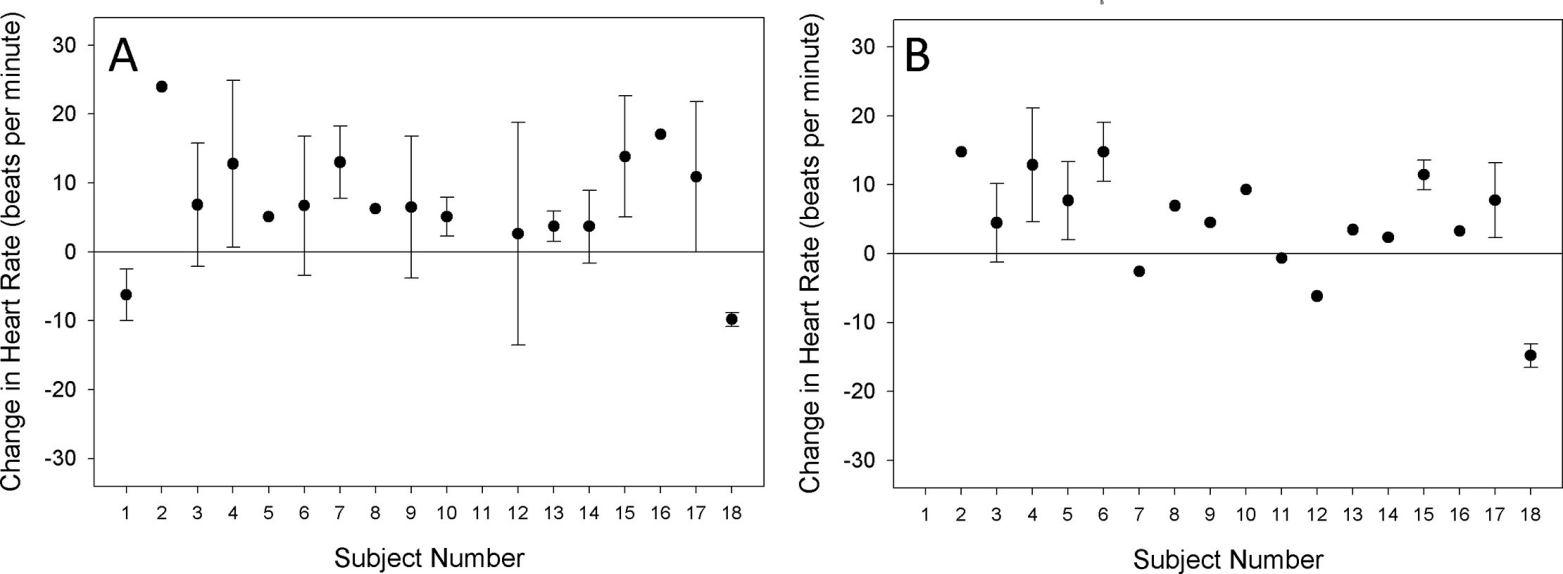
Mean differences in observed HR (SD) across individual’s events (some only had one event) as compared to baseline during non-exam EMA-reported stressors (panel A) and exams reported by participants during screening (panel B).

Fig 3 shows the individual differences in mean HR estimates from baseline for 18 participants during non-exam EMA-reported stressors and exams reported during screening (corresponding to comparisons C2 and C5 respectively in Table 1). These data show that for most individuals, the HR was elevated during periods of stress with the magnitude of the increase varying considerably between individuals. A similar significant elevation in HR was observed during the verbal fluency cognitive task (comparison C6: 7.1 bpm) as compared to the stress-free baseline (F(1,659929) = 78.7, p<0.001). During the mental arithmetic task (comparison C7), HR was not elevated (F(1,660030) = 0.90, p = 0.34).

There was no significant correlation observed between mean stressfulness of events self-reported via EMA surveys and mean change in HR from baseline during the events (rho = .11; p = .50). We also examined the correlation between the change in heart rate during exam periods reported during screening (comparison C5) and self-reported anticipated stressfulness for those exams and found no correlation (rho = .002, p = .99).

## Discussion

We have developed and pilot-tested an informatics framework that uses multiple commercial and public platforms and APIs to collect and process physiological data from consumer wearable fitness tracker devices measuring the physiological response to psychosocial stress. Prior literature shows that mild psychosocial stress elicits a measurable HR response on the order or 5–10 bpm [22]. Our preliminary results indicate that low-cost consumer activity trackers such as Fitbit® may be used to measure HR response to everyday stressors as well as standardized stressors. We also provide initial data regarding the feasibility of conducting studies of stress exposure in everyday life completely remotely with existing technology, which is particularly important in the context of conducting large-scale population surveillance studies. Future larger studies are needed to more fully evaluate the strengths and limitations of this approach.

As the purpose of this study was to determine the feasibility of remotely measuring an individual’s response to stressful situation in the naturalistic environment, use of a reference device was not feasible. We therefore relied on an examination of concurrent validity of the Fitbit® HR sensors by using events known to be generally stressful (i.e., college examinations) and other events self-reported by participants. Thus, while we were not able to determine how accurately Fitbit® HR sensors measure true heart rate, we were able to confirm that HR measurements obtained with Fitbit® increase as expected and consistently in response to naturalistic stressors (in the absence of physical activity) and physical activity across multiple people and multiple events. Furthermore, the magnitude of the increase is consistent with that obtained in laboratory conditions– 5–10 beats per minute [22,49]. These results indicate that a) the elevation in HR due to stressful life events is detectable with consumer wearable devices in the natural environment, and b) there is substantial inter-participant variability in HR changes which is also consistent with prior findings showing that individuals vary in their physiological reactivity to stress [50]. Our results also indicate that unlike the VF task, the remotely administered mental arithmetic task we used does not elicit a stress response (albeit the former is based on a smaller sample). Prior work on heart rate that included verbal fluency tasks shows that verbal fluency is a mildly stressful cognitive task [51]. The lack of a robust response in our study to the mental arithmetic task may be due to a number of factors including the telephone response system implementation of the task which does not have a verbal component that is a requirement of the verbal fluency tasks.

We also found that subjective self-report of the severity of event stressfulness are not reflective of the physiological response measured via heart rate. This is consistent with prior reports in the literature of the lack of a strong relationship between subjective self-assessments of acute mental stress and objective measures [52,53].

Our study has a number of limitations that should be considered in the interpretation of the findings. Since this is a pilot feasibility study, the sample size is limited to 18 young adult, mostly female college students. A larger study with a greater number of participants and greater gender and age distribution range would be helpful to confirm our findings and to conduct a more in-depth analysis of the impact of individual differences on the ability to detect heart rate changes with consumer fitness trackers. Another limitation is that in this study, we focused on a single tracker device brand (Fitbit®) capable of measuring heart rate. Our findings may therefore not be readily generalizable to other devices nor could we distinguish (due to small sample size) if there are differences among the various Fitbit® devices used by participants in this study. Another limitation of using Fitbit® as the wearable device tested is that the Fitbit® API does not provide access to inter-beat interval data from the optical HR sensor. This data would have been necessary in order to estimate heart rate variability (HRV), a measure shown to be responsive to psychosocial stress [54]. Other limitations include not having estimates of alcohol intake that may potentially affect heart rate measurements and not being able to determine if the participant was exerting themselves while remaining stationary (e.g., lifting heavy objects or other sources of stationary physical strain).

Nonetheless, this study showed that despite these limitations, heart rate response to stressful events encountered in everyday life can be measured using a widely used, commercially available device. Future studies assessing devices with additional capabilities (for example, those that allow for the calculation of HRV or for assessing electrodermal activity) may further improve on the ability to detect stressful events and overcome the additional limitation in the current study that heart rate increases can occur due to multiple factors other than stress (that include among others physical activity, mood, food consumption and smoking [55]. In the current pilot study, we excluded measures associated with activity and did not attempt to account for other factors.

## Conclusions

We have developed an informatics framework that uses multiple commercial and public platforms and APIs to collect and process physiological data from consumer wearable fitness tracker devices. Using this framework, we found that widely available and accessible consumer wearable fitness trackers such as Fitbit® with HR sensor capabilities are able to capture changes in continuous heart rate in response to naturally occurring psychosocial stressors. These findings serve as a foundation to further explore the use of commercially available wearable devices for quantifying the burden of stress in everyday life and its association with health outcomes. More work is needed to determine the most effective way of measuring physiological response to stress in naturalistic environments; however, the results of this pilot study provide an initial indication that it is feasible to continually monitor for potential stress exposure and to assess one’s reactivity to a standardized stressor remotely.

## References

[1] Kivimäki M, Steptoe A. Effects of stress on the development and progression of cardiovascular disease. Nature Reviews Cardiology. 2017;15: 215–229. 10.1038/nrcardio.2017.189 29213140

[2] Hackett RA, Steptoe A. Type 2 diabetes mellitus and psychological stress—a modifiable risk factor. Nat Rev Endocrinol. 2017;13: 547–560. 10.1038/nrendo.2017.64 28664919

[3] Sinha R, Jastreboff A. Stress as a common risk factor for obesity and addiction. Biol Psychiatry. 2013;73: 827–835. 10.1016/j.biopsych.2013.01.032 23541000 PMC3658316

[4] Han KS, Kim L, Shim I. Stress and sleep disorder. Exp Neurobiol. 2012;21: 141–150. 10.5607/en.2012.21.4.141 23319874 PMC3538178

[5] Kim E-J, Dimsdale JE. The Effect of Psychosocial Stress on Sleep: A Review of Polysomnographic Evidence. Behavioral Sleep Medicine. 2007;5: 256–278. 10.1080/15402000701557383 17937582 PMC4266573

[6] Madsen IEH, Nyberg ST, Magnusson Hanson LL, Ferrie JE, Ahola K, Alfredsson L, et al Job strain as a risk factor for clinical depression: systematic review and meta-analysis with additional individual participant data. Psychol Med. 2017;47: 1342–1356. 10.1017/S003329171600355X 28122650 PMC5471831

[7] O’Donnell MJ, Chin SL, Rangarajan S, Xavier D, Liu L, Zhang H, et al Global and regional effects of potentially modifiable risk factors associated with acute stroke in 32 countries (INTERSTROKE): a case-control study. Lancet. 2016;388: 761–775. 10.1016/S0140-6736(16)30506-2 27431356

[8] Airagnes G, Lemogne C, Goldberg M, Hoertel N, Roquelaure Y, Limosin F, et al Job exposure to the public in relation with alcohol, tobacco and cannabis use: Findings from the CONSTANCES cohort study. Sanchez ZM, editor. PLOS ONE. 2018;13: e0196330 10.1371/journal.pone.0196330 29715268 PMC5929509

[9] Hyman SM, Sinha R. Stress-related factors in cannabis use and misuse: implications for prevention and treatment. J Subst Abuse Treat. 2009;36: 400–413. 10.1016/j.jsat.2008.08.005 19004601 PMC2696937

[10] Preston KL, Kowalczyk WJ, Phillips KA, Jobes ML, Vahabzadeh M, Lin J-L, et al Context and craving during stressful events in the daily lives of drug-dependent patients. Psychopharmacology (Berl). 2017;234: 2631–2642. 10.1007/s00213-017-4663-0 28593441 PMC5709189

[11] Sinha R. How does stress increase risk of drug abuse and relapse? Psychopharmacology (Berl). 2001;158: 343–359. 10.1007/s002130100917 11797055

[12] Torres OV, O’Dell LE. Stress is a principal factor that promotes tobacco use in females. Prog Neuropsychopharmacol Biol Psychiatry. 2016;65: 260–268. 10.1016/j.pnpbp.2015.04.005 25912856 PMC4618274

[13] Justice NJ. The relationship between stress and Alzheimer’s disease. Neurobiol Stress. 2018;8: 127–133. 10.1016/j.ynstr.2018.04.002 29888308 PMC5991350

[14] Rosengren A, Hawken S, Ounpuu S, Sliwa K, Zubaid M, Almahmeed WA, et al Association of psychosocial risk factors with risk of acute myocardial infarction in 11119 cases and 13648 controls from 52 countries (the INTERHEART study): case-control study. Lancet. 2004;364: 953–962. 10.1016/S0140-6736(04)17019-0 15364186

[15] Smyth A, O’Donnell M, Lamelas P, Teo K, Rangarajan S, Yusuf S. Physical Activity and Anger or Emotional Upset as Triggers of Acute Myocardial InfarctionClinical Perspective: The INTERHEART Study. Circulation. 2016;134: 1059–1067. 10.1161/CIRCULATIONAHA.116.023142 27753614

[16] Wilbert-Lampen U, Leistner D, Greven S, Pohl T, Sper S, Völker C, et al Cardiovascular Events during World Cup Soccer. New England Journal of Medicine. 2008;358: 475–483. 10.1056/NEJMoa0707427 18234752

[17] Brotman DJ, Golden SH, Wittstein IS. The cardiovascular toll of stress. Lancet. 2007;370: 1089–1100. 10.1016/S0140-6736(07)61305-1 17822755

[18] Chrousos GP. Stress and disorders of the stress system. Nat Rev Endocrinol. 2009;5: 374–381. 10.1038/nrendo.2009.106 19488073

[19] Foley P, Kirschbaum C. Human hypothalamus–pituitary–adrenal axis responses to acute psychosocial stress in laboratory settings. Neuroscience & Biobehavioral Reviews. 2010;35: 91–96. 10.1016/j.neubiorev.2010.01.010 20109491

[20] Jacobs SC, Friedman R, Parker JD, Tofler GH, Jimenez AH, Muller JE, et al Use of skin conductance changes during mental stress testing as an index of autonomic arousal in cardiovascular research. American Heart Journal. 1994;128: 1170–1177. 10.1016/0002-8703(94)90748-x 7985598

[21] Kop WJ, Weissman NJ, Zhu J, Bonsall RW, Doyle M, Stretch MR, et al Effects of acute mental stress and exercise on inflammatory markers in patients with coronary artery disease and healthy controls. Am J Cardiol. 2008;101: 767–773. 10.1016/j.amjcard.2007.11.006 18328837

[22] Kotlyar M, Brauer LH, al’absi M, Adson DE, Robiner W, Thuras P, et al Effect of bupropion on physiological measures of stress in smokers during nicotine withdrawal. Pharmacol Biochem Behav. 2006;83: 370–379. 10.1016/j.pbb.2006.02.017 16581115

[23] Kotlyar M, al’Absi M, Brauer LH, Grant JE, Fong E, Kim SW. Naltrexone effect on physiological and subjective response to a cold pressor task. Biological Psychology. 2008;77: 233–236. 10.1016/j.biopsycho.2007.10.005 18053632 PMC2758556

[24] Kotlyar M, al’Absi M, Thuras P, Vuchetich JP, Adson DE, Nowack AL, et al Effect of paroxetine on physiological response to stress and smoking. Psychosom Med. 2013;75: 236–243. 10.1097/PSY.0b013e3182898f6d 23504241 PMC3721515

[25] Kotlyar M, Lindgren BR, Vuchetich JP, Le C, Mills AM, Amiot E, et al Timing of nicotine lozenge administration to minimize trigger induced craving and withdrawal symptoms. Addictive Behaviors. 2017;71: 18–24. 10.1016/j.addbeh.2017.02.018 28235705 PMC5449230

[26] Steptoe A, Hamer M, Chida Y. The effects of acute psychological stress on circulating inflammatory factors in humans: a review and meta-analysis. Brain Behav Immun. 2007;21: 901–912. 10.1016/j.bbi.2007.03.011 17475444

[27] Vinkers CH, Penning R, Hellhammer J, Verster JC, Klaessens JHGM, Olivier B, et al The effect of stress on core and peripheral body temperature in humans. Stress. 2013;16: 520–530. 10.3109/10253890.2013.807243 23790072

[28] Fava GA, McEwen BS, Guidi J, Gostoli S, Offidani E, Sonino N. Clinical characterization of allostatic overload. Psychoneuroendocrinology. 2019;108: 94–101. 10.1016/j.psyneuen.2019.05.028 31252304

[29] McEwen BS. Physiology and Neurobiology of Stress and Adaptation: Central Role of the Brain. Physiological Reviews. 2007;87: 873–904. 10.1152/physrev.00041.2006 17615391

[30] McEwen BS. From serendipity to clinical relevance: How clinical psychology and neuroscience converged to illuminate psychoneuroendocrinology. Psychoneuroendocrinology. 2019;105: 36–43. 10.1016/j.psyneuen.2018.09.011 30309685

[31] McEwen BS, Wingfield JC. The concept of allostasis in biology and biomedicine. Hormones and Behavior. 2003;43: 2–15. 10.1016/s0018-506x(02)00024-7 12614627

[32] Sala M, Brosof LC, Rosenfield D, Fernandez KC, Levinson CA. Stress is associated with exercise differently among individuals with higher and lower eating disorder symptoms: An ecological momentary assessment study. Int J Eat Disord. 2017;50: 1413–1420. 10.1002/eat.22799 29098699 PMC5761745

[33] Shiffman S, Stone AA, Hufford MR. Ecological Momentary Assessment. Annual Review of Clinical Psychology. 2008;4: 1–32. 10.1146/annurev.clinpsy.3.022806.091415 18509902

[34] Trull TJ, Ebner-Priemer UW. Using experience sampling methods/ecological momentary assessment (ESM/EMA) in clinical assessment and clinical research: Introduction to the special section. Psychological Assessment. 2009;21: 457–462. 10.1037/a0017653 19947780 PMC4255457

[35] Everson SA, Lynch JW, Chesney MA, Kaplan GA, Goldberg DE, Shade SB, et al Interaction of workplace demands and cardiovascular reactivity in progression of carotid atherosclerosis: population based study. BMJ. 1997;314: 553–558. 10.1136/bmj.314.7080.553 9055713 PMC2126071

[36] Danielsen A, Olofsen H, Bremdal BA. Increasing fall risk awareness using wearables: A fall risk awareness protocol. Journal of Biomedical Informatics. 2016;63: 184–194. 10.1016/j.jbi.2016.08.016 27544413

[37] Kennedy AP, Epstein DH, Jobes ML, Agage D, Tyburski M, Phillips KA, et al Continuous in-the-field measurement of heart rate: Correlates of drug use, craving, stress, and mood in polydrug users. Drug Alcohol Depend. 2015;151: 159–166. 10.1016/j.drugalcdep.2015.03.024 25920802 PMC4447529

[38] Rahman MdM, Epstein DH, Preston KL, Jobes M, Beck JG, Kedia S, et al Are we there yet?: feasibility of continuous stress assessment via wireless physiological sensors. Proceedings of the 5th ACM Conference on Bioinformatics, Computational Biology, and Health Informatics—BCB ‘14. Newport Beach, California: ACM Press; 2014 pp. 479–488. 10.1145/2649387.2649433 PMC4374173 25821861

[39] Sprint G, Cook DJ, Schmitter-Edgecombe M. Unsupervised detection and analysis of changes in everyday physical activity data. J Biomed Inform. 2016;63: 54–65. 10.1016/j.jbi.2016.07.020 27471222 PMC11323554

[40] Kumar S, Abowd GD, Abraham WT, al’Absi M, Gayle Beck J, Chau DH, et al Center of excellence for mobile sensor data-to-knowledge (MD2K). Journal of the American Medical Informatics Association. 2015;22: 1137–1142. 10.1093/jamia/ocv056 26555017 PMC5009911

[41] Sano A, Taylor S, McHill AW, Phillips AJ, Barger LK, Klerman E, et al Identifying Objective Physiological Markers and Modifiable Behaviors for Self-Reported Stress and Mental Health Status Using Wearable Sensors and Mobile Phones: Observational Study. J Med Internet Res. 2018;20: e210 10.2196/jmir.9410 29884610 PMC6015266

[42] Sano A, Phillips AJ, Yu AZ, McHill AW, Taylor S, Jaques N, et al Recognizing academic performance, sleep quality, stress level, and mental health using personality traits, wearable sensors and mobile phones 2015 IEEE 12th International Conference on Wearable and Implantable Body Sensor Networks (BSN). Cambridge, MA, USA: IEEE; 2015 pp. 1–6. 10.1109/BSN.2015.7299420 PMC5431072 28516162

[43] Umematsu T, Sano A, Picard RW. Daytime Data and LSTM can Forecast Tomorrow’s Stress, Health, and Happiness 2019 41st Annual International Conference of the IEEE Engineering in Medicine and Biology Society (EMBC). Berlin, Germany: IEEE; 2019 pp. 2186–2190. 10.1109/EMBC.2019.8856862 31946335

[44] Harris PA, Taylor R, Thielke R, Payne J, Gonzalez N, Conde JG. Research electronic data capture (REDCap)—A metadata-driven methodology and workflow process for providing translational research informatics support. Journal of Biomedical Informatics. 2009;42: 377–381. 10.1016/j.jbi.2008.08.010 18929686 PMC2700030

[45] Kirschbaum C, Pirke KM, Hellhammer DH. The ‘Trier Social Stress Test’—a tool for investigating psychobiological stress responses in a laboratory setting. Neuropsychobiology. 1993;28: 76–81. 10.1159/000119004 8255414

[46] Abeare CA, Freund S, Kaploun K, McAuley T, Dumitrescu C. The Emotion Word Fluency Test (EWFT): Initial psychometric, validation, and physiological evidence in young adults. Journal of Clinical and Experimental Neuropsychology. 2017;39: 738–752. 10.1080/13803395.2016.1259396 27892775

[47] Marek-Spartz K, Knoll B, Bill R, Christie T, Pakhomov S. System for Automated Speech and Language Analysis (SALSA) Proceedings of the Annual Conference of the International Speech Communication Association. Singapore: International Speech and Communication Association; 2014 pp. 2142–2143.

[48] Pakhomov SVS, Teeple W, Mills AM, Kotlyar M. Use of an automated mobile application to assess effects of nicotine withdrawal on verbal fluency: A pilot study. Exp Clin Psychopharmacol. 2016;24: 341–347. 10.1037/pha0000089 27690503 PMC5065262

[49] Shcheslavskaya OV, Burg MM, McKinley PS, Schwartz JE, Gerin W, Ryff CD, et al Heart rate recovery after cognitive challenge is preserved with age. Psychosom Med. 2010;72: 128–133. 10.1097/PSY.0b013e3181c94ca0 20028831 PMC2950633

[50] Larson MR, Ader R, Moynihan JA. Heart Rate, Neuroendocrine, and Immunological Reactivity in Response to an Acute Laboratory Stressor: Psychosomatic Medicine. 2001;63: 493–501. 10.1097/00006842-200105000-00020 11382278

[51] Kennedy DO, Scholey AB. Glucose administration, heart rate and cognitive performance: effects of increasing mental effort. Psychopharmacology (Berl). 2000;149: 63–71. 10789884 10.1007/s002139900335

[52] Campbell J, Ehlert U. Acute psychosocial stress: Does the emotional stress response correspond with physiological responses? Psychoneuroendocrinology. 2012;37: 1111–1134. 10.1016/j.psyneuen.2011.12.010 22260938

[53] Hellhammer J, Schubert M. The physiological response to Trier Social Stress Test relates to subjective measures of stress during but not before or after the test. Psychoneuroendocrinology. 2012;37: 119–124. 10.1016/j.psyneuen.2011.05.012 21689890

[54] Kim H-G, Cheon E-J, Bai D-S, Lee YH, Koo B-H. Stress and Heart Rate Variability: A Meta-Analysis and Review of the Literature. Psychiatry Investig. 2018;15: 235–245. 10.30773/pi.2017.08.17 29486547 PMC5900369

[55] Fatisson J, Oswald V, Lalonde F. Influence Diagram of Physiological and Environmental Factors Affecting Heart Rate Variability: An Extended Literature Overview. Heart International. 2016;11: heartint.500023. 10.5301/heartint.5000232 27924215 PMC5056628

